# Sequential Bone Repair in Rabbit Sinus Lifts Using Bio-Oss and Hyaluronic Acid–Polynucleotide Gel (Regenfast)

**DOI:** 10.3390/jfb15120361

**Published:** 2024-11-28

**Authors:** Nozomi Maniwa, Samuel Porfirio Xavier, Sergio Luis Scombatti de Souza, Erick Ricardo Silva, Daniele Botticelli, Kenzo Morinaga, Shunsuke Baba

**Affiliations:** 1 Department of Oral Implantology, School of Dentistry, Osaka Dental University, 8-1 Kuzuhahanazonocho, Hirakata 573-1121, Osaka, Japan; nmani19850426@gmail.com (N.M.); morinaga-k@cc.osaka-dent.ac.jp (K.M.); baba-s@cc.osaka-dent.ac.jp (S.B.); 2 Department of Oral and Maxillofacial Surgery and Periodontology, Faculty of Dentistry of Ribeirão Preto, University of São Paulo, Av. do Café-Subsetor Oeste-11 (N-11), Ribeirão Preto 14040-904, SP, Brazil; spx@forp.usp.br (S.P.X.); scombatti@forp.usp.br (S.L.S.d.S.); erick.silva@usp.br (E.R.S.); 3 ARDEC Academy, 47923 Rimini, Italy

**Keywords:** animal study, bone healing, histology, bone augmentation, Regenfast, biomaterial, bone defect

## Abstract

Background: A comprehensive investigation to associate the use of polynucleotides and hyaluronic acid with bovine bone in maxillary sinus lift procedures in rabbits has not been performed yet. The aim was to evaluate the influence of this novel association on the processes of bone regeneration in maxillary sinus augmentation. Methods: In this prospective, randomized, within-animal model, maxillary sinus augmentation was performed bilaterally in 12 rabbits. Deproteinized bovine bone material (DBBM) was used as filler material. A hyaluronic acid–polynucleotide gel was aggregated at the test site. Histological evaluations were performed after 2 and 10 weeks of healing. Results: After 2 weeks of healing, similar amounts of new bone were observed at both the control (7.7 ± 4.3%) and test sites (8.1 ± 3.8%; *p* = 0.697). Bone formation was observed predominantly along the osteotomy margins and adjacent sinus walls. After 10 weeks of healing, the total new bone fraction reached 28.0 ± 10.1% at the control sites and 27.3 ± 10.5% at the test sites (*p* = 0.563). Multiple perforations of the sinus mucosa were observed in both groups when in contact with the biomaterial granules. Conclusion: The present study failed to demonstrate a positive effect on bone formation when incorporating hyaluronic acid–polynucleotide gel (Regenfast) into a xenograft for maxillary sinus augmentation. Additionally, the use of this gel did not mitigate the occurrence of thinned mucosa or reduce the risk of subsequent sinus mucosa perforation.

## 1. Introduction

The presence of adequate bone volume is imperative for the successful placement of implants, significantly influencing long-term outcomes [1]. In instances where sufficient bone volume is absent in the posterior maxilla, sinus floor elevation has been established as a reliable surgical technique that facilitates oral rehabilitation through implant placement. Among the various approaches, lateral access is frequently employed for sinus lifting [2,3].

However, extensive research has demonstrated that, in the absence of implants or biomaterials within the elevated sinus space, the sinus mucosa is prone to revert to its original position [4,5]. To address this challenge, a range of grafting materials has been utilized to fill the augmented maxillary sinus. Notably, xenogeneic bone granules derived from diverse animal species have been extensively documented in the literature [6]. Among these materials, deproteinized bovine bone mineral (DBBM) processed at a low temperature of 300 °C has been employed in numerous clinical [7,8,9,10,11,12] and animal studies [4,5,13,14]. This particular xenogeneic bone exhibits a slow resorption rate and possesses commendable osteoconductive properties [13,14,15].

In addition to these materials, recent therapeutic strategies have focused on enhancing the healing process itself, particularly through the stimulation of fibroblast functionality [16]. Polynucleotides (PN) have been shown to augment cell numbers in human fibroblast cultures while also enhancing protein synthesis and promoting the expression of collagen types I and III, thereby improving wound healing both in vitro and in vivo [17]. These polynucleotides are classified as a gel-based medical device comprising salified chains of deoxyribonucleic acids [17].

While increased collagen production may indicate enhanced natural tissue repair—potentially significant for clinical applications such as filling contracted or depressed areas—there is a critical concern regarding the risk of fibrosis associated with excessive collagen deposition. Fibrosis can disrupt the normal physiological tissue structure, leading to functional and morphological impairments [18]; however, it has been confirmed that PN not only fosters an environment conducive to collagen deposition but also indirectly accelerates tissue recovery without exhibiting signs of fibrosis [17].

Alongside PN, high-molecular-weight hyaluronic acid has emerged as a promising wound-healing agent owing to its bacteriostatic, anti-inflammatory, and immunosuppressive properties [19]. Hyaluronic acid is involved in various signaling pathways activated during the wound healing process and may facilitate re-epithelialization [20]. Moreover, hyaluronic acid enhances the proliferation and migration of human oral fibroblasts and periodontal ligament cells [21,22].

Pre-clinical studies evaluating bone regeneration in surgically created defects have demonstrated that the combination of autogenous bone, collagen sponge, or xenografts with hyaluronic acid results in increased bone formation and reduced residual graft material [23,24,25]. In a study conducted by Kim et al. [26], chronic pathology was induced in extraction sockets, and the application of hyaluronic acid resulted in accelerated wound healing and enhanced bone formation. Similarly, Hammad et al. reported that hyaluronic acid promotes the rapid closure of wound margins in excisional palatal wounds [27].

Recent pre-clinical investigations have further illustrated the beneficial effects of hyaluronic acid on periodontal regeneration [28] and root coverage [29]. Notably, hyaluronic acid appears to stimulate angiogenesis [30] and support bone repair in the short term [31]. When combined with L-PRF, hyaluronic acid has been associated with superior soft tissue healing [32], reduced edema, and decreased analgesic consumption [33].

Moreover, clinical studies concerning non-surgical periodontal therapy [34,35,36,37,38,39], regenerative periodontal surgery [40,41], and root coverage [42] have consistently demonstrated superior clinical outcomes when hyaluronic acid is employed as an adjunct to conventional methods. Additionally, promising clinical outcomes have been reported for hyaluronic acid when utilized as a filler for papillary reconstruction [43,44,45,46].

A randomized controlled trial (RCT) recently highlighted how the use of hyaluronic acid can speed up palatal wound healing and alleviate postoperative pain following connective tissue graft harvesting [47]. Similarly, another RCT reported that hyaluronic acid, when applied during laser-assisted frenectomy, also enhanced wound healing and reduced postoperative discomfort [48]. Several studies collectively support the efficacy of hyaluronic acid in promoting wound repair by enhancing granulation tissue formation, reducing excessive inflammation during healing, and encouraging both re-epithelialization and angiogenesis [49]. Additionally, a meta-analysis revealed that the non-surgical use of hyaluronic acid significantly improved clinical attachment levels and probing depth [38].

To the best of our knowledge, no thorough study has yet examined the combined use of polynucleotides, hyaluronic acid, and bovine bone in maxillary sinus lift procedures in rabbits. Through histological and histomorphometric evaluations, this research aims to investigate how this innovative combination impacts bone regeneration during maxillary sinus augmentation.

## 2. Materials and Methods

### 2.1. Ethical Statements

This research project was approved by the Ethics Committee on Animal Use at the Faculty of Dentistry of Ribeirão Preto, University of São Paulo CEUA, Brazil, on 14 March 2023 (protocol #0066/2023). The experimental procedures were conducted in full compliance with Brazilian legislation on animal experimentation. Additionally, the ARRIVE guidelines were followed throughout the study (Supplementary Materials).

### 2.2. Study Design

In this prospective, randomized, within-animal model (test and control sides in the same animal), maxillary sinus augmentation was performed bilaterally. Deproteinized bovine bone material (DBBM) was used as filler material. A hyaluronic acid–polynucleotide gel was aggregated at the test site. Histological evaluations were performed after 2 and 10 weeks of healing.

### 2.3. Experimental Animals and Sample Size

No prior data were available to estimate the minimum sample size required; however, it was considered that a 5 ± 2.5% difference in new bone formation across the total area after a 10-week healing period would justify the treatment. Based on a two-tailed test with α = 0.05 and a power of 0.9, a sample size of 5 animal pairs was calculated to reject the null hypothesis of no significant difference (PS Power and Sample Size Calculations) [50]. To account for potential experimental complications, the sample size was increased to 6 pairs. Consequently, twelve adult male New Zealand White rabbits, evenly distributed between the two healing periods, were selected for the study, weighing 3.5–4.0 kg and aged 5–6 months.

### 2.4. Randomization and Allocation Concealment

Randomization and allocation concealment were managed electronically by an author (S.P.X.) who did not participate in the animal selection or surgical procedures. Treatment assignments were kept confidential in sealed, opaque envelopes, which were only opened right before the placement of the first graft. The histological examiner was blinded to both the healing time and the treatment allocation, with the histological slides coded accordingly. Nevertheless, the specific characteristics of the sites were still distinguishable in the histological slides.

### 2.5. Biomaterials

Bio-Oss is an osteoconductive, slowly resorbed xenogeneic bone substitute derived from the mineral portion of bovine bone. It undergoes a deproteinization process, resulting in a highly porous, inorganic structure that closely resembles human cancellous bone in terms of physical and chemical composition [51].

Regenfast is a combination of hyaluronic acid combined with highly purified and completely resorbable polynucleotides aiming to support the regeneration of oral tissues [21].

### 2.6. Anesthetic Procedures

The surgical procedures were carried out under general anesthesia, initiated with an intramuscular injection of acepromazine (1.0 mg/kg; Acepran, Vetnil, Louveira, São Paulo) in combination with xylazine (3.0 mg/kg; Dopaser^®^, Hertape Calier, Juatuba, Minas Gerais, Brazil), and ketamine hydrochloride (50.0 mg/kg; União Química Farmacêutica Nacional S/A, Embuguaçú, São Paulo, Brazil), administered 15 min after the acepromazine. Once the animals reached an appropriate level of sedation, they received a prophylactic antibiotic dose of oxytetracycline (0.2 mL/kg; Biovet, Vargem Grande Paulista, São Paulo, Brazil), meloxicam 0.2%, 1.0 mg/kg subcutaneously (Flamavet; União Química Farmacêutica S/A, Embu-Guaçu, São Paulo, Brazil), and tramadol hydrochloride 5.0 mg/kg subcutaneously (Halexistar; Goiânia, Goiás, Brazil). The surgical site was shaved and disinfected using a 1% polyvinylpyrrolidone iodine solution (Riodeíne Tincture, Rioquímica, São José do Rio Preto, São Paulo, Brazil). Local anesthesia was provided through the application of 2% mepivacaine with 1:100,000 norepinephrine (Mepinor, Nova DFL, Rio de Janeiro, Brazil).

### 2.7. Surgical Procedure

**Figure 1:**
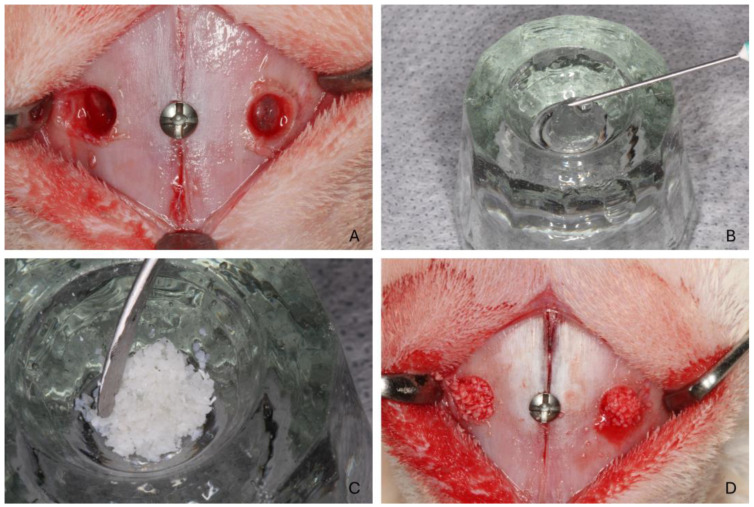
Clinical procedures. (**A**) Osteotomies were prepared laterally to the naso-incisal suture. A small screw was placed to mark the center of the osteotomies for subsequent histological analysis. (**B**) Regenfast was dispensed into a glass dappen dish; (**C**) Bio-Oss^®^ granules were mixed with Regenfast; (**D**) After the elevation of the sinus mucosa, the grafts were inserted into the elevated space.

All surgical procedures were conducted by a single operator (V.F.B.; see acknowledgments) with extensive experience and expertise. A 2.0 cm incision was made along the nasal dorsum’s midline, followed by meticulous dissection through the muscle layers to expose the periosteum and nasal bone. Osteotomies were performed 1.0 cm anterior to the naso-frontal suture and 4 mm lateral to the naso-incisal suture on both sides. Bilateral maxillary sinus openings, each 4.0 mm in diameter, were created using #6 spherical diamond drills, following the protocol established by the research group (Figure 1A). A small reference screw was placed at the naso-incisal suture to center the osteotomies for subsequent histological assessments. The sinus membrane was elevated carefully using maxillary sinus curettes (Bontempi^®^, Bmed Srl, San Giovanni in Marignano, Italy).

Each sinus was filled with 0.1 cc (100 mm^3^) of Bio-Oss^®^ (0.1 cc, Geistlich, Switzerland). Based on the pre-established randomization, one side of the maxillary sinus was grafted with a combination of Regenfast^®^ (0.1 mL, Mastelli, Italy; Figure 1B) mixed with biomaterial particles (Figure 1C), while the contralateral side received Bio-Oss^®^ (0.1 cc) alone as the control. The biomaterial was carefully placed inside the elevated space (Figure 1D). The periosteum was closed using resorbable sutures (Polyglactin 910 5–0, Vicryl^®^, Ethicon, Johnson & Johnson, São José dos Campos, Brazil), and the skin was sutured with nylon (Ethilon 4–0^®^, Ethicon).

### 2.8. Animal Maintenance

Anti-inflammatory medications, subcutaneous meloxicam 0.2%, 0.5 mg/kg (Flamavet; União Química Farmacêutica S/A, Embu-Guaçu, São Paulo, Brazil), and subcutaneous tramadol hydrochloride 5.0 mg/kg (Halexistar; Goiânia, Goiás, Brazil), were maintained for the first three days post-operation and administered once a day.

The animals were kept at the Animal Facility of the Faculty of Dentistry, University of São Paulo, Ribeirão Preto campus. They were housed individually in metal cages (one animal per 4500 cm^2^) within a temperature-controlled environment (20–22 °C, 50% humidity), equipped with air conditioning and an exhaust system ensuring 27–34 air exchanges per hour. The lighting was automatically regulated to maintain a 12-h light/dark cycle. The rabbits were provided with a tailored diet and had unlimited access to water. A strict monitoring protocol was followed throughout the experiment, with daily checks of basic physiological functions, food intake, excretory habits, and behavioral indicators of postoperative pain. Post-surgical infections, wound care, suture integrity, and any bleeding or signs of infection were also closely monitored.

### 2.9. Euthanasia

Euthanasia was carried out through an intravenous overdose of thiopental (2.0 mL, 1.0 g; Thiopentax, Cristália, Itapira, São Paulo, Brazil) after 2 or 10 weeks, with 10 animals allocated to each group. The experimental sites were surgically retrieved, segmented into individual blocks, and fixed in 10% paraformaldehyde for preservation.

### 2.10. Histological Processing

The specimens were prepared for histological analysis at the hard tissue section laboratory of FORP-USP. They were first rinsed under running water to remove any residual fixation solution completely, then dehydrated using a series of increasing ethyl alcohol concentrations. This sequence involved constant agitation and alcohol changes every three days (60%, 80%, 96%, and two rounds of absolute alcohol). The samples were then embedded in resin (LR White™ HardGrid, London Resin Co., Ltd., Berkshire, United Kingdom) for impregnation and later polymerized in an oven at 60 °C.

After polymerization, each block was sectioned along the transaxial plane, centered by the fixation screw placed during the surgery to mark the midpoint of the graft.

Two sections, approximately 100–150 µm thick, were produced using precision cutting and grinding equipment (Exakt, Apparatebau, Norderstedt, Germany) and then further reduced to a thickness of 60–80 µm. The histological sections were stained using either Toluidine Blue or a combination of Stevenel’s Blue and Alizarin Red.

### 2.11. Histomorphometric Evaluation

**Figure 2:**
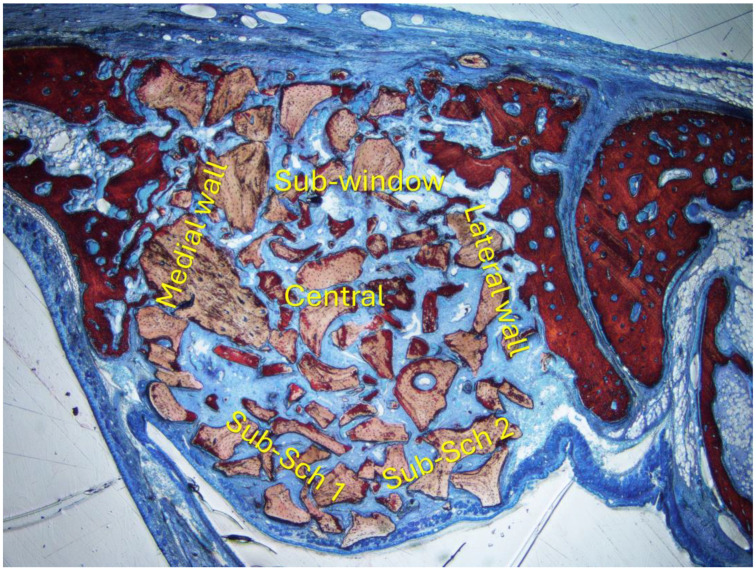
Six areas were evaluated: sub-Schneiderian (2 areas), medial and lateral walls, central, and sub-window.

Histological and histomorphometric analyses were conducted by an expert evaluator (E.R.S.) who was calibrated with another specialist (S.P.X.) until achieving a Cohen’s kappa coefficient exceeding 0.90. Imaging was performed using an Eclipse Ci optical microscope (Nikon Corporation, Tokyo, Japan) paired with a Digital Sight DS-2Mv camera (Nikon Corporation, Tokyo, Japan) connected to a computer. Histological images were analyzed at ×100 magnification with the NIS-Elements software 4.1 (Nikon, Tokyo, Japan). A lattice grid of 1200 µm × 900 µm in dimension with squares of 75 µm was superimposed on the images, and the following areas were evaluated: sub-Schneiderian (2 areas), medial and lateral walls, central, and sub-window (Figure 2). The following tissue types were quantified within each area: new bone, xenograft material, and soft tissue. The percentages of each tissue type were calculated relative to the total area.

Additionally, the number and size of sinus mucosa perforations in contact with biomaterial granules, as well as areas of adhesion between the elevated mucosa and the pristine mucosa, were evaluated using a 20× objective (approximately ×200 magnification).

Finally, thin mucosa regions (<40 µm), along with the pristine mucosa and the dimensions of the associated pseudostratified epithelium, were assessed using a 40× objective (approximately ×400 magnification).

### 2.12. Data Analysis

The results are presented as mean ± standard deviation. The primary variable for histomorphometric analysis was new bone formation, while secondary variables included other tissue types. The analysis also considered the number of perforations and thin mucosa sites in the elevated mucosa. The Shapiro–Wilk test was employed to assess the normality of these data. Based on the normality results, differences between test and control sites were analyzed using either a paired *t*-test or a Wilcoxon matched-pairs signed-rank test. Statistical analyses were performed using GraphPad Prism (version 10 for Windows, GraphPad Software, Boston, MA, USA). A significance level of 5% was applied for all tests.

## 3. Results

### 3.1. Clinical Outcomes 

The healing of the animals was uneventful. All histological slides were available for analysis, with n = 6 for both periods.

### 3.2. Descriptive Histological Evaluation

**Figure 3:**
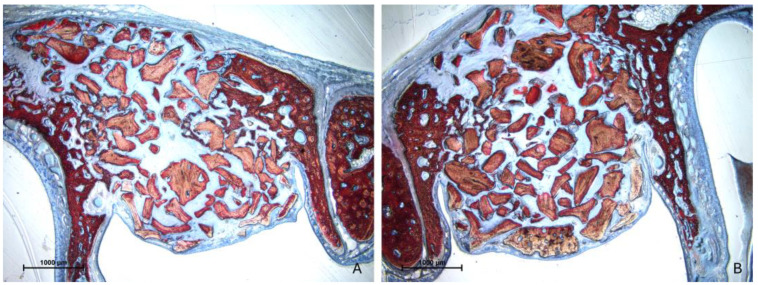
Photomicrographs of ground sections after 2 weeks of healing. (**A**) test site (Regenfast mixed to Bio-Oss^®^ granules); (**B**) control site (only Bio-Oss^®^ granules). The histological characteristics were comparable between the test and control groups, presenting new bone close to the bone window and very little in the other regions. Stevenel’s blue and alizarin red stain.

After two weeks of healing, the histological characteristics were comparable between the test and control groups (Figure 3A,B). 

**Figure 4:**
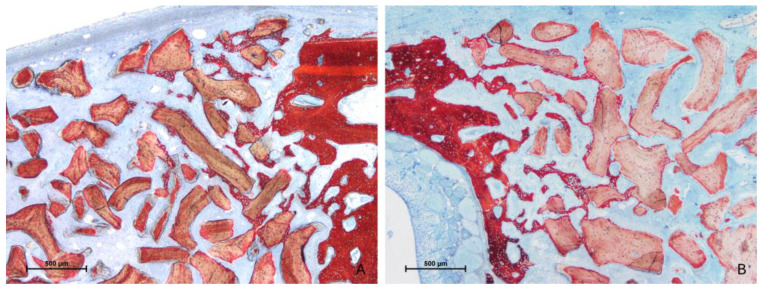
Photomicrographs of ground sections after 2 weeks of healing illustrate bone formation close to the osteotomy margins and adjacent to the sinus walls. (**A**) test site; (**B**) control site. Stevenel’s blue and alizarin red stain.

New bone formation was observed predominantly along the osteotomy margins and adjacent to the sinus walls (Figure 4A,B). 

**Figure 5:**
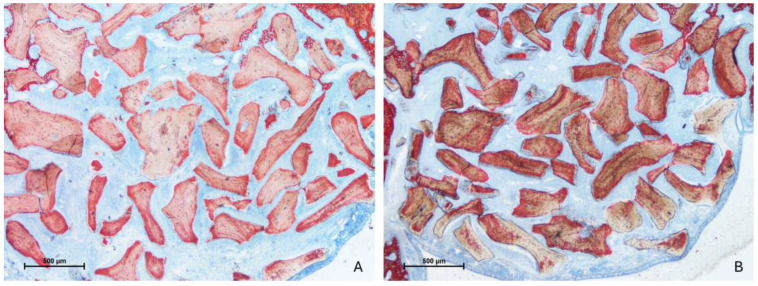
Photomicrographs of ground sections after 2 weeks of healing. The regions near the center and the sub-Schneiderian area showed minimal new bone formation, with graft granules predominantly encapsulated by soft tissues. (**A**) test site; (**B**) control site. Stevenel’s blue and alizarin red stain.

However, in other areas, only limited new bone formation was noted, with the elevated space largely filled by soft tissues and residual xenograft material (Figure 5A,B). 

**Figure 6:**
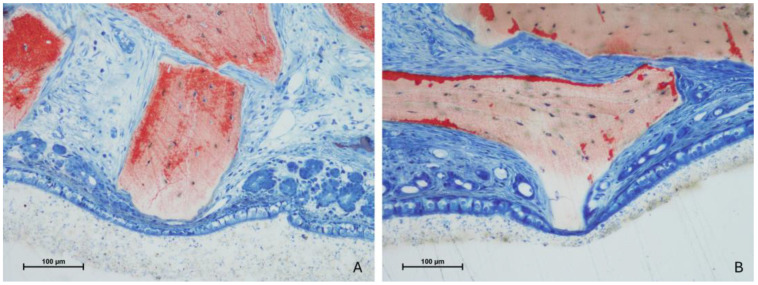
Photomicrographs of ground sections after 2 weeks of healing. (**A**) Test site. The graft granule in contact with the sinus mucosa displaced the mucosal glands and vessels, leading to a reduction in mucosal thickness. (**B**) Control site. In more advanced cases, the lamina propria was absent, leaving only a thinned pseudostratified epithelium Stevenel’s blue and alizarin red stain.

Importantly, no perforations of the sinus mucosa related to the presence of xenograft granules were observed. Nevertheless, several sites exhibited thinning of the mucosa. In these regions, the biomaterial granules displaced mucosal glands and vessels, leading to a reduction in mucosal thickness (Figure 6A). In more advanced cases, the lamina propria was absent, leaving only a thinned pseudostratified epithelium (Figure 6B). 

**Figure 7:**
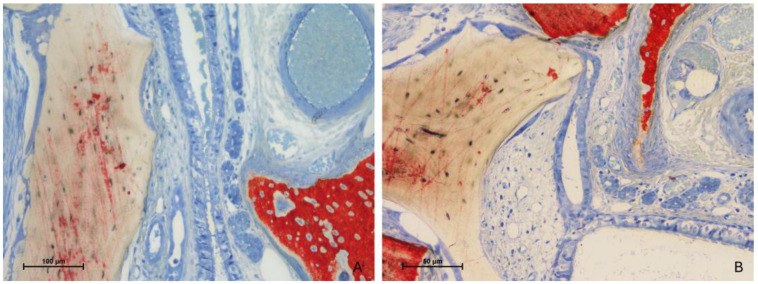
Photomicrographs of ground sections after 2 weeks of healing. Adhesion processes were evident where the elevated mucosa made contact with the pristine mucosa still attached to the bone wall. (**A**) Test site. Proximity stage. The two epithelia were in close contact, with the cilia intertwining and traces of mucus present between them. (**B**) Control site. The epithelia of the elevated and pristine mucosa fused, attempting to restore the area. Stevenel’s blue and alizarin red stain.

Adhesion processes were evident where the elevated mucosa made contact with the pristine mucosa still attached to the bone wall. Both proximity (Figure 7A) and fusion stages (Figure 7B) were identified, though no synechiae were observed.

**Figure 8:**
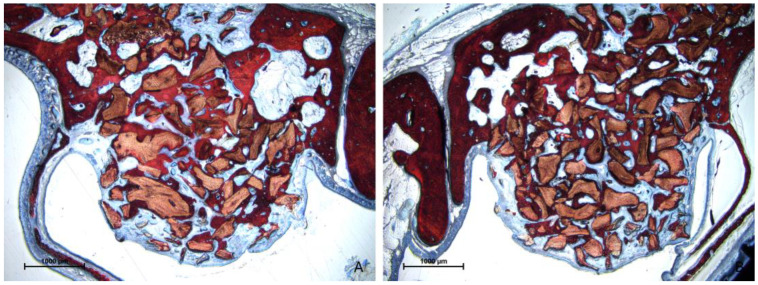
Photomicrographs of ground sections after 10 weeks of healing. The histological characteristics were comparable between the test and control groups. A notable increase in new bone formation was observed in all evaluated regions compared with the 2-week period. (**A**) test site (Regenfast mixed to Bio-Oss^®^ granules); (**B**) control site (only Bio-Oss^®^ granules). Stevenel’s blue and alizarin red stain.

After 10 weeks of healing, there was a notable increase in new bone formation in all evaluated regions (Figure 8A,B).

**Figure 9:**
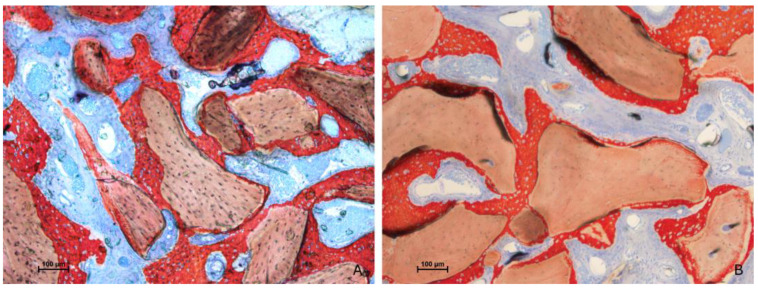
Photomicrographs of ground sections after 10 weeks of healing. Bone growth was observed extending along the granule surfaces, forming bridges between them and utilizing the osteoconductive potential of the biomaterial. (**A**) test site; (**B**) control site. Stevenel’s blue and alizarin red stain.

Bone growth was observed extending along the granule surfaces, forming bridges between them and utilizing the osteoconductive potential of the biomaterial (Figure 9A,B).

**Figure 10:**
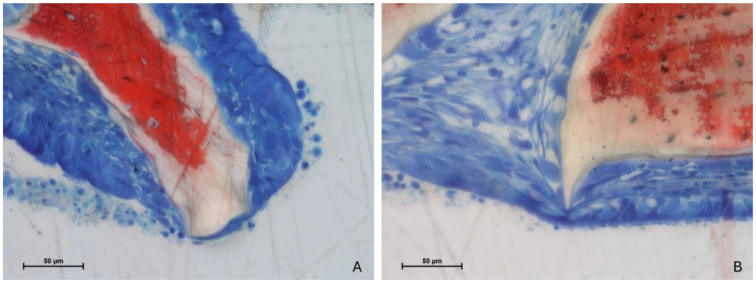
Photomicrographs of ground sections after 10 weeks of healing. Thinned mucosa sites corresponding to the granules were observed in both groups. (**A**) Test site. The sinus mucosa appears almost perforated. (**B**) Control site. Sharp tips or irregular ridges of the non-resorbable biomaterial may have contributed to mucosal thinning and the progression to perforation. Stevenel’s blue and alizarin red stain.

**Figure 11:**
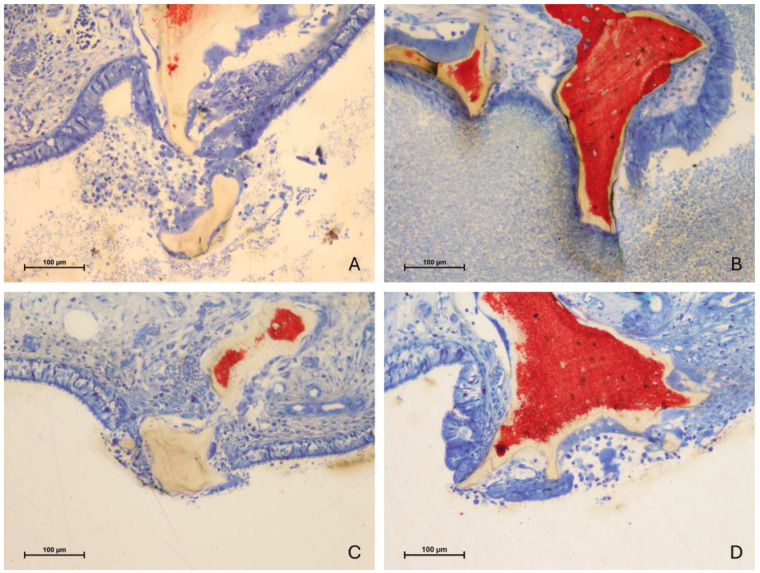
Photomicrographs of ground sections after 10 weeks of healing. Perforations of the sinus mucosa caused by contact with granules were observed in both groups. The granules were frequently associated with inflammatory infiltrates and macrophage-like cells. The epithelial cells appeared to attempt to encapsulate the graft, protecting the inner portion of the elevated space. (**A**,**B**) test sites. Note the exudate within the sinus. (**C**,**D**) control sites. Stevenel’s blue and alizarin red stain.

Despite this, areas of thinned mucosa (Figure 10A,B) and perforations (Figure 11A–D) corresponding to the granules were observed in both groups, particularly near sharp tips or irregular ridges of the non-resorbable biomaterial. Additionally, a few adhesion processes were identified, though they appeared minimal.

### 3.3. Histomorphometric Assessments 

After two weeks of healing, the total new bone fraction was 7.7 ± 4.3% at the control sites and 8.1 ± 3.8% at the test sites, with no statistically significant difference between the two (Table 1). 

The majority of new bone was located along the bone walls, with values of 12.7 ± 8.4% and 17.6 ± 6.3% at the control and test sites, respectively, though again, these differences were not statistically significant. Higher percentages of xenograft material and lower percentages of soft tissue were observed at the control sites compared with the test sites across all examined regions. This difference reached statistical significance only in the sub-Schneiderian region.

By 10 weeks of healing, one animal showed an infiltrate within both sinuses; however, this condition did not affect bone formation, being the total amount similar to the mean values of the respective group. The amount of new bone had increased across all regions (Table 2), with no significant differences between the groups.

The proportion of xenograft material had decreased compared with the 2-week time point in both groups. The differences compared with the previous period of examination were statistically significant for both new bone and xenograft percentages in both test and control sites. Consistently, higher percentages of xenograft and lower percentages of soft tissue were noted at the control sites relative to the test sites in all examined regions.

### 3.4. Mucosa Assessments 

The thickness of the pristine sinus mucosa ranged from 127 µm to 172 µm on average (Table 3).

At the 2-week time point, 11 sites of thinned mucosa (<40 µm) were observed in both the control and test groups, with no perforations detected in either group. After 10 weeks of healing, the number of thinned mucosa sites increased to 30 at the test sites and 10 at the control sites. The minimum width observed for the thinned mucosa was 3 µm. Several perforations of the sinus mucosa in proximity to the xenograft granules were observed, with 5 perforations at the control sites and 11 at the test sites. These perforations were found in 4 out of 6 sinuses in both groups.

The mean width of the pseudostratified epithelium of the pristine mucosa range was 28–38 µm (Table 4). The width in the thinned mucosa sites ranged between 14 and 17 µm, with a minimum value of 3 µm.

## 4. Discussion

The aim of the present study was to evaluate the influence of incorporating a hyaluronic acid–polynucleotide gel into a xenograft on bone formation when used as a filler material for maxillary sinus augmentation. The findings from this study did not demonstrate any significant effect of Regenfast on bone formation at either of the examined time points. Specifically, no differences in the percentages of new bone formation were observed between the test sites, where the gel was added, and the control sites, where only the xenograft was used without the gel. It is important to note that the xenograft was less represented in the test sites, likely because of the volume occupied by the gel combined with the xenograft prior to placement in the augmented space. The increase in new bone between the two healing periods indicates that the presence of Regenfast neither contributed to nor hindered bone formation. Additionally, the xenograft percentage decreased between the two healing periods in both groups, suggesting that a certain degree of resorption occurred during the initial healing phase. The extrusion of some biomaterial granules through the antrostomy cannot be excluded.

One possible explanation for the lack of a positive effect on bone regeneration when using polynucleotide and hyaluronic acid gel is related to its primary mechanism of action. Evidence suggests that polynucleotides may primarily enhance fibroblast activity, collagen synthesis, and tissue repair rather than directly stimulating bone formation [17]. This aligns with findings demonstrating increased production of Col1a1 and Col3a1 in treated tissues, which are essential for wound healing and extracellular matrix organization but do not necessarily translate to osteogenic activity. Thus, while the gel might support soft tissue healing and collagen maturation, its potential to influence bone regeneration appears limited, particularly in this experimental context.

However, the granules of biomaterial used in this study once again demonstrated their excellent properties, particularly their ability to maintain the volume achieved following sinus augmentation over time and their conductivity, which facilitates the spread of new bone from the sinus walls towards the central and sub-Schneiderian regions. In the latter region, specifically, no new bone formation was observed after the initial 2-week healing period. The potential of the sinus mucosa to contribute to bone formation has been clearly demonstrated in several studies [52,53,54,55,56]; however, this potential has been questioned in in vivo situations [57]. Additionally, it has been shown that the structure of the pseudo-periosteum beneath the sinus mucosa is lost during the early stages of healing following sinus augmentation [58].

In the present study, the impact of the contact between the granules and the sinus mucosa was evaluated. It was observed that this contact initially caused the displacement of local structures, such as glands and vessels, followed by thinning of the sinus mucosa, which may lead over time to its perforation. Indeed, several instances of thinned mucosa were noted at both healing periods, while perforations were only observed at the 10-week mark. This finding suggests that perforation is a result of progressive thinning of the sinus mucosa and is not attributable to the surgical procedure itself. Instead, it appears to be caused by the pressure within the sinus as it tends to regain its original volume. The sinus mucosa is gradually pushed against the granules, leading to the thinning process, which is exacerbated by the shape of the granules, particularly the presence of peaks and ridges. This outcome was first identified in a previous rabbit study [59] and has been further supported by other experiments [60,61]. Similar effects were also caused by the apex and threads of implants [60,62]. This complication is strongly linked to the type of material used, with a lower incidence observed when autogenous bone or collagenated grafts are employed [60,61]. It is important to note that rabbit sinus mucosa is thinner (Table 3; range 127–172 µm) compared with human mucosa. In a clinical study involving eighty-eight patients scheduled for sinus floor elevation [63], the mean sinus mucosa thickness was 2.0 mm; however, thirty patients had a mean mucosa thickness of ≤1 mm, and in four patients, it was ≤0.5 mm. Given the progressive nature of this condition, as demonstrated in the present study, the use of xenografts that may induce such effects should be carefully considered in clinical practice. Although no complications related to this phenomenon have been documented thus far, patients should be informed that granules may be expelled through the nose at any time after sinus floor elevation, even though this occurrence is unlikely to pose a significant health concern.

Another noteworthy observation was the detection of adhesion phenomena, which have been previously described in other studies [64]. Following sinus augmentation, the elevated sinus mucosa can come into contact with the pristine, non-elevated mucosa still attached to the sinus walls, either during surgery or as a result of subsequent edema. This contact can develop into an adhesion, which may further progress to a fusion stage, where the epithelial cells of the elevated and non-elevated sinus mucosa interpenetrate or fuse with one another. In more advanced cases, this can evolve into synechia, where the lamina propria of both mucosae become bridged together.

In the present study, two distinct healing periods were analyzed. This approach aimed to assess whether Regenfast could enhance bone formation during the early phases. Additionally, evaluating sinus mucosa damage across two consecutive periods helped rule out early perforations potentially caused by surgical complications.

The limitations of the present study are related to the animal model used and to the low sample size that experimental studies often use to comply with the 3Rs principles. Nevertheless, the present experimental study provided evidence of a possible effect on bone formation after sinus augmentation of a hyaluronic acid–polynucleotide gel applied together with a bovine xenograft. In addition to the limitations mentioned, experimental studies often face challenges in replicating the exact clinical conditions found in human patients. Furthermore, the controlled environments in which these studies are conducted may not fully capture the complexity of real-world clinical scenarios, where factors such as patient variability, comorbidities, and environmental influences play a significant role. Finally, while experimental studies allow for precise control over variables, they generally lack the long-term follow-up necessary to assess the longevity and stability of the treatment outcomes.

## 5. Conclusions

The present study failed to demonstrate a positive effect on bone formation when incorporating hyaluronic acid–polynucleotide gel (Regenfast) into a xenograft for maxillary sinus augmentation. Additionally, the use of this gel did not mitigate the occurrence of thinned mucosa or reduce the risk of subsequent sinus mucosa perforation.

## Figures and Tables

**Table 1:** Histomorphometric measurements in the various areas were analyzed after 2 weeks of healing. Mean percentage values ± standard deviation. *, *p* < 0.05.

2 Weeks	Bio-Oss Alone			Bio-Oss Regenfast		
	New Bone	Xenograft	Soft Tissue	New Bone	Xenograft	Soft Tissue
Sub-Schneiderian	4.4 ± 2.8	45.7 ± 8.8 *	49.9 ± 9.6 *	4.6 ± 3.8	37.7 ± 3.8 *	57.7 ± 4.6 *
Walls	12.7 ± 8.4	46.9 ± 7.5	40.4 ± 7.6	17.6 ± 6.3	36.2 ± 8.7	46.3 ± 8.7
Central	7.4 ± 8.3	43.2 ± 7.6	49.4 ± 13.3	3.8 ± 3.7	41.0 ± 14.4	55.1 ± 12.7
Sub-Window	6.4 ± 5.2	40.4 ± 10.9	53.2 ± 13.2	6.4 ± 5.9	38.3 ± 10.1	55.3 ± 6.1
Total	7.7 ± 4.3	44.0 ± 6.2	48.2 ± 8.9	8.1 ± 3.8	38.3 ± 7.4	53.6 ± 5.6

**Table 2:** Histomorphometric measurements in the various areas were analyzed after 10 weeks of healing. Mean percentage values ± standard deviation. *, *p* < 0.05.

10 Weeks	Bio-Oss Alone			Bio-Oss Regenfast		
	New Bone	Xenograft	Soft Tissue	New Bone	Xenograft	Soft Tissue
Sub-Schneiderian	27.6 ± 27.6	34.2 ± 7.8 *	38.2 ± 14.3 *	25.3 ± 11.9	24.3 ± 10.8 *	50.5 ± 16.0 *
Walls	29.9 ± 10.0	29.4 ± 13.9	40.7 ± 22.5	27.6 ± 8.4	26.7 ± 11.4	45.7 ± 16.3
Central	27.9 ± 10.8	35.5 ± 7.5 *	36.5 ± 12.8	28.0 ± 12.8	22.2 ± 14.7 *	49.9 ± 23.8
Sub-Window	26.5 ± 14.2	31.8 ± 12.6	41.7 ± 23.9	28.3 ± 16.6	22.7 ± 9.4	49.0 ± 24.1
Total	28.0 ± 10.1	32.7 ± 7.6 *	39.3 ± 16.8	27.3 ± 10.5	24.0 ± 9.6 *	48.8 ± 18.0

**Table 3:** Width of the pristine mucosa; number, mean width ± standard deviation, number of sites, and minimum width of thinned mucosae; number and dimensions of perforations and sinuses involved after 2 and 10 weeks.

		Pristine	Thinned Mucosae			Perforations		
			No	Mean	Min	No	Dimension	Sinus
2 weeks	Bio-Oss alone	127 ± 33	11	25 ± 4	3	0	NA	0
	Bio-Oss Regenfast	138 ± 54	11	24 ± 12	3	0	NA	0
10 weeks	Bio-Oss alone	172 ± 43	30	20 ± 4	3	5	297 ± 390	4
	Bio-Oss Regenfast	130 ± 30	10	19 ± 8	5	11	158 ± 101	4

**Table 4:** Width of the pseudostratified epithelium at the pristine site; number, mean width ± standard deviation, minimum width of the pseudostratified epithelium in the thinned sites.

		Pristine	Epithelial Cells in the Thinned Mucosa	
			Mean	Min
2 weeks	Bio-Oss alone	28 ± 4	16 ± 5	3
	Bio-Oss Regenfast	33 ± 6	14 ± 7	3
10 weeks	Bio-Oss alone	38 ± 5	17 ± 3	3
	Bio-Oss Regenfast	34 ± 4	17 ± 7	5

## Data Availability

The original contributions presented in the study are included in the article; further inquiries can be directed to the corresponding author.

## Supplementary Materials

The following supporting information can be downloaded at: https://www.mdpi.com/article/10.3390/jfb15120361/s1.
